# Goldilocks Mastectomy: The Middle Road Option for Obese Breast Cancer Patients

**DOI:** 10.7759/cureus.50362

**Published:** 2023-12-11

**Authors:** Chai Wei Tong, Ruben Cohen-Hallaleh

**Affiliations:** 1 Surgery, St George Hospital, Kogarah, AUS; 2 Surgery, Sydney Surgical, Sydney, AUS

**Keywords:** skin-sparing mastectomy, breast reconstruction, wise pattern, general surgery and breast cancer, oncoplastic, goldilocks mastectomy

## Abstract

Breast cancer is the predominant cancer affecting females in Australia. With the rising obesity rates, the number of obese breast cancer patients is also rising. Full complex breast reconstruction is known to carry significant risk of complications in obese patients, hence we sought to utilize Goldilocks mastectomy as an alternative middle-ground option between standard mastectomy and complex breast reconstruction.

A 63-year-old obese female presented with left nipple inversion. Ultrasonography revealed a 9.7 mm dilated retroareolar duct within the left breast, indicating lobular carcinoma. Subsequent biopsy analysis revealed estrogen/progesterone receptor (ER/PR)-positive cancer with equivocal human epidermal growth factor receptor 2 (HER2) status and a Ki67 index of 10%. Concurrently, a discrete area of conspicuous breast tissue prominence displayed characteristics of invasive ductal carcinoma with similar receptor status but a reduced Ki67 index of less than 5%. Traditional breast reconstruction options were unfavorable due to obesity, prompting consideration of a tailored Goldilocks mastectomy. The procedure was uncomplicated, and follow-up assessments revealed commendable wound healing, alongside the restoration of symmetrical breast contours.

Conclusion: This study highlights the significance of Goldilocks mastectomy as an invaluable technique in the comprehensive management of breast cancer, particularly for obese patients characterized by a body mass index (BMI) exceeding 35 and substantial medical comorbidities.

## Introduction

Breast cancer is the most common cancer among females in Australia and is the second most common cancer diagnosed in Australia [1]. Concurrently, obesity rates have almost doubled in the last 20 years; this means that we are likely to see more obese patients with breast cancer [2]. Autologous and implant-based reconstructions are not recommended for these patients due to their increased risk of failure and surgical complications [3,4]. The Goldilocks mastectomy, introduced in 2012, offers an innovative alternative for patients who are at high risk or prefer not to undergo conventional methods of breast reconstruction. This approach is a single-step procedure, which entails conducting a skin-sparing mastectomy, utilizing Wise pattern incisions, and forming a breast mound using the remaining lower mastectomy flap [5]. Here we present a case of a 63-year-old female diagnosed with breast cancer, for whom traditional breast reconstruction was not a viable option due to her obesity. Consequently, she underwent a successful and uncomplicated Goldilocks mastectomy, with a contralateral breast reduction to achieve symmetrical breasts, all within a single operation.

## Case presentation

A 63-year-old obese female presented for investigation of left nipple inversion. Ultrasonography revealed a 9.7 mm dilated retroareolar duct within the left breast, replete with internal debris. Subsequent biopsy analysis revealed the presence of lobular carcinoma, characterized by estrogen receptor (ER) and progesterone receptor (PR) positivity, equivocal human epidermal growth factor receptor 2 (HER2) status, and a Ki67 index of 10%. Concurrently, a discrete area of conspicuous breast tissue prominence, situated at the 10 o'clock position, approximately 5 cm from the nipple, displayed strong posterior acoustic shadowing on ultrasound. Histological examination of the biopsied tissue confirmed the presence of invasive ductal carcinoma, similarly demonstrating ER/PR positivity, and equivocal HER2 status, albeit with a reduced Ki67 index of less than 5%.

The patient underwent further consultation with a breast surgeon and plastic surgeon to discuss mastectomy options. It is noteworthy that she did not want a standard mastectomy. Unfortunately, her obesity rendered traditional breast reconstructive options, including therapeutic mammoplasty, implant-based reconstruction, and free flap procedures, untenable due to elevated susceptibility to adverse outcomes, including tissue necrosis and infection.

**Figure 1:**
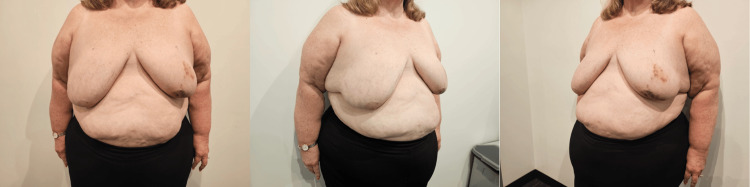
Pre-operative photos showing breast cancer on the left breast.

**Figure 2:**
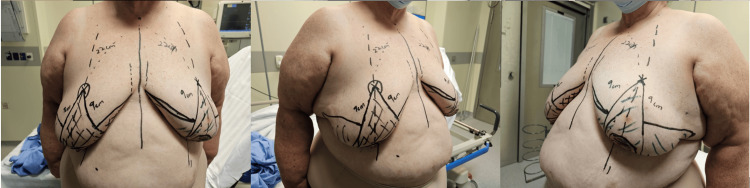
Pre-operative Wise pattern incision markings. The apex of the reduction pattern is positioned along the breast meridian projecting forward from the level of the inframammary fold. Vertical limbs are then drawn at an 80-degree angle. The medial and lateral horizontal lines are then drawn.

**Figure 3:**
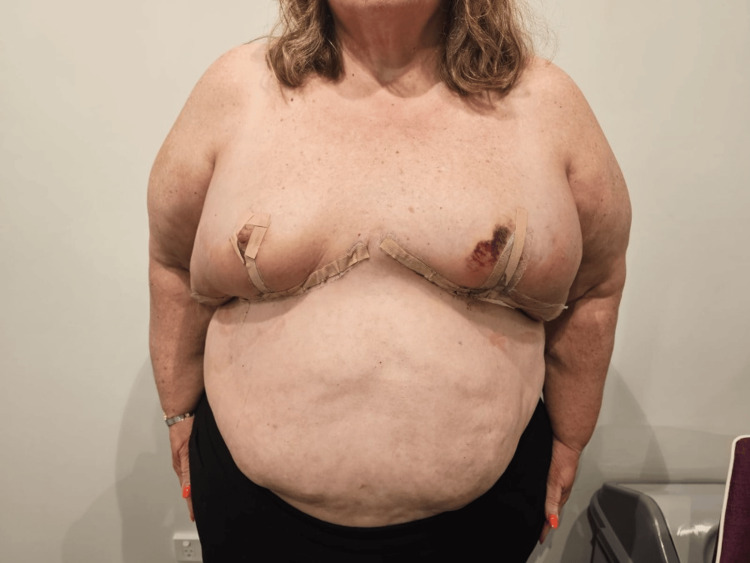
Immediate post-operative photo showing symmetrical breast contours.

To address this complex predicament, a tailored approach involving a Goldilocks mastectomy was offered to her. Figure 1 shows the pre-operative photos and Figure 2 shows the pre-operative incision markings made at standing position. The procedure went smoothly without complications in the immediate post-operative period (Figure 3).

**Figure 4:**
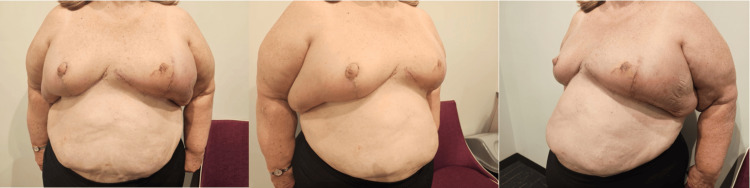
Progress photos at three weeks post-operatively demonstrating good wound healing and preservation of symmetrical breast contours.

**Figure 5:**
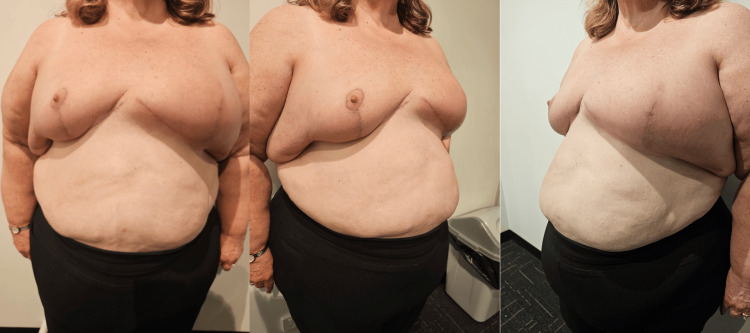
Progress photos at two months post-operatively demonstrating ongoing good wound healing and preservation of symmetrical breast contours.

**Figure 6:**
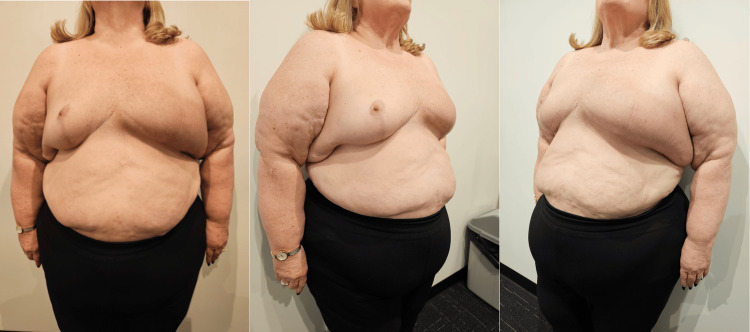
Progress photos at six months post-operatively demonstrating healed wounds and preservation of symmetrical breast contours.

Subsequent follow-up assessments at three weeks, two months, and six months post-operatively, as pictured in Figures 4, 5, 6, revealed commendable wound healing and the restoration of symmetrical breast contours.

## Discussion

Goldilocks mastectomy is a form of skin-sparing mastectomy with a Wise pattern incision, followed by the formation of a breast mound using residual inferior mastectomy flap tissue. This technique was first described in 2012 by Richardson and Ma. The name is inspired by the fairytale character Goldilocks, in that this technique offers a more esthetic outcome compared to conventional mastectomy but at the same time avoids the complexity of formal breast reconstruction [5]. Despite the introduction of this technique a decade ago, there have only been small numbers of single-institution studies and case reports demonstrating the application and outcomes of this technique.

The surgical steps can be outlined as follows. Wise pattern markings are made pre-operatively with the patient standing. The apex of the reduction pattern is positioned along the breast meridian projecting forward from the level of the inframammary fold. Vertical limbs are then drawn at an 80-degree angle. The medial and lateral horizontal lines are then drawn (Figure 2). These lines serve as a guide for the incisions. Skin-sparing mastectomy is then performed through either a circumareolar incision or an elliptical incision. All visible breast tissues are removed while preserving perfusion to the skin flaps. To create a breast mound, the lower pole fasciocutaneous flaps are first de-epithelialized within the boundaries of the Wise pattern incision. This is followed by a separation of the upper and lower poles of the breast. The lower pole tissue is then repositioned and sutured on the chest wall in a mound-like shape. The skin is closed using the standard Wise pattern by suturing the inferior end of the upper pole to the de-epithelialized dermis of the lower pole [5].

The key benefit of Goldilocks mastectomy is that it is a single-step procedure, avoiding the need for a second procedure and hence reducing the potential complications of a second surgery as well as implant or flap-related complications [5]. Patients with medical comorbidities such as obesity, BMI >35, diabetes, and immunosuppression are known to have higher risks of developing complications in autologous and implant-based breast reconstruction [3,6]. The Goldilocks mastectomy is thus a suitable alternative to breast reconstruction for the patient. Furthermore, the Goldilocks mastectomy has also been considered a safe option for patients with high-risk thrombosis requiring immediate post-operative anticoagulation, as described by Schwartz and Skowronski [7].

As with any skin-sparing mastectomies, the Goldilocks technique removes all breast tissue preserving as much skin as possible along with the inframammary fold. The difference between Goldilocks mastectomy and other skin-sparing mastectomies is that immediate breast reconstruction is achieved using local residual mastectomy flaps rather than implant or distant flaps. Therefore, the Goldilocks technique is better suited for women with larger breasts or breast ptosis, who will have sufficient local soft tissue to re-create the breast mound [8]. Smaller-breasted women may necessitate a second-stage operation for skin tailoring, fat grafting, or implant placement to achieve an optimal outcome [9]. Schwartz proposed the addition of the lateral intercostal artery perforator (LICAP) flap to Goldilocks mastectomy as a single-stage procedure for massive weight loss patients to further augment the volume of the residual mastectomy flaps; the safety of this combined approach was supported by a small case series demonstrating no complications requiring take-back to theatre [10,11]. While there is no large-scale, long-term data on the oncological safety of Goldilocks mastectomy, this should be similar to any other skin-sparing mastectomy technique. A recent Cochrane review concluded that the overall survival and local recurrence-free survival were similar between skin-sparing mastectomy and conventional mastectomy, although this was based on very low-certainty evidence [12].

Further advancements have allowed the preservation of the nipple-areolar complex using a dermal pedicle or a Skin-sparing, Wise patterns, Internal Mammary perforator (SWIM) flap [13-15]. Goldilocks mastectomy has also been employed as a baseline for secondary breast reconstruction procedures and as a salvage option for failed reconstructions or as a bridge to secondary reconstructive procedures [8]. Unfortunately, nipple-sparing procedures were not an option for her due to the location of her tumor.

## Conclusions

Goldilocks mastectomy is not only a surgical technique but a tailored solution between standard mastectomy and conventional breast reconstruction. It addresses the needs and considerations of patients with obesity and significant medical comorbidities. This study serves as yet another descriptive example of the effectiveness and value of Goldilocks mastectomy, and we hope to see an increase in the application of this technique in enhancing outcomes and patient satisfaction in the realm of breast cancer treatment.
